# Supplementary material for: Excitonic structure and pumping power dependent emission blue-shift of type-II quantum dots
Source: Sci Rep. 2017 Mar 30;7:45568. doi: 10.1038/srep45568 (PMC5372090; doi:10.1038/srep45568)
Supplement: Supplementary Information [file srep45568-s1.pdf]

Excitonic structure and pumping power dependent emission blue-shift of type-II quantum dots

Supplementary Information

Petr Klenovský,[a] Petr Steindl, and Dominique Geffroy
Central European Institute of Technology, Masaryk University, Kamenice 753/5, 62500 Brno, Czech Republic and
Department of Condensed Matter Physics, Faculty of Science, Masaryk University, Kotlářská 2, 61137 Brno, Czech Republic

SI. CI IMPLEMENTATION

We describe here our implementation of the CI technique. The multi-particle Slater determinants of the theory are made up of single-particle wavefunctions $\psi(\mathbf{r})$ determined using the envelope function approximation, based on the 8-band $\mathbf{k}\cdot\mathbf{p}$ formalism:

$$\psi_{a_i}(\mathbf{r}) = \sum_{\nu\in\{s,x,y,z\}\bigotimes\{\uparrow,\downarrow\}} \chi_{a_i,\nu}(\mathbf{r})u_\nu^\Gamma(\mathbf{r}), \tag{1}$$

where s corresponds to the conduction band Bloch wave and x, y, and z correspond to the Bloch waves of the valence band, which are antisymmetric with respect to the corresponding mirror plane; $\uparrow$ and $\downarrow$ indicate the spin; $\chi_{a_i,\nu}(\mathbf{r})$ stands for the envelope function, $a\in\{e,h\}$ where e stands for the electron and h for hole, respectively. $e_i\in\{1\dots n_e\}$ ($h_i\in\{1\dots n_h\}$), where n_e (n_h) is the number of single-particle electron (hole) states considered in our full configuration interaction approach. The many-body problem is solved variationally in the subspace spanned by the Slater determinants made up from these wavefunctions. The expressions for the Slater determinants depend on the total number of particles in the system, and, for a given total number of particles, on the specific number of electrons and holes considered. To be specific, we give below the form of the trial wavefunctions for all cases considered in this work. For the neutral exciton X^0, we have

$$\left|X^0\right\rangle = \sum_{i=1}^{n_e}\sum_{j=1}^{n_h}\eta_{ij}\begin{vmatrix}\psi_{ei}(\mathbf{r}_e) & \psi_{ei}(\mathbf{r}_h)\\ \psi_{hj}(\mathbf{r}_e) & \psi_{hj}(\mathbf{r}_h)\end{vmatrix}, \tag{2}$$

for the positive trion X^+

$$\left|X^+\right\rangle = \sum_{i=1}^{n_e}\sum_{\substack{j,k=1\\k>j}}^{n_h}\eta^+_{ijk}\begin{vmatrix}\psi_{ei}(\mathbf{r}_e) & \psi_{hj}(\mathbf{r}_e) & \psi_{hk}(\mathbf{r}_e)\\ \psi_{ei}(\mathbf{r}_{h1}) & \psi_{hj}(\mathbf{r}_{h1}) & \psi_{hk}(\mathbf{r}_{h1})\\ \psi_{ei}(\mathbf{r}_{h2}) & \psi_{hj}(\mathbf{r}_{h2}) & \psi_{hk}(\mathbf{r}_{h2})\end{vmatrix}, \tag{3}$$

for the negative trion X^-

$$\left|X^-\right\rangle = \sum_{\substack{i,j=1\\j>i}}^{n_e}\sum_{k=1}^{n_h}\eta^-_{ijk}\begin{vmatrix}\psi_{ei}(\mathbf{r}_{e1}) & \psi_{ej}(\mathbf{r}_{e1}) & \psi_{hk}(\mathbf{r}_{e1})\\ \psi_{ei}(\mathbf{r}_{e2}) & \psi_{ej}(\mathbf{r}_{e2}) & \psi_{hk}(\mathbf{r}_{e2})\\ \psi_{ei}(\mathbf{r}_{h}) & \psi_{ej}(\mathbf{r}_{h}) & \psi_{hk}(\mathbf{r}_{h})\end{vmatrix}, \tag{4}$$

and for the neutral biexciton XX^0

$$\left|XX^0\right\rangle = \sum_{\substack{i,j=1\\j>i}}^{n_e}\sum_{\substack{k,l=1\\k>l}}^{n_h}\eta^{XX}_{ijkl}\begin{vmatrix}\psi_{ei}(\mathbf{r}_{e1}) & \psi_{ej}(\mathbf{r}_{e1}) & \psi_{hk}(\mathbf{r}_{e1}) & \psi_{hl}(\mathbf{r}_{e1})\\ \psi_{ei}(\mathbf{r}_{e2}) & \psi_{ej}(\mathbf{r}_{e2}) & \psi_{hk}(\mathbf{r}_{e2}) & \psi_{hl}(\mathbf{r}_{e2})\\ \psi_{ei}(\mathbf{r}_{h1}) & \psi_{ej}(\mathbf{r}_{h1}) & \psi_{hk}(\mathbf{r}_{h1}) & \psi_{hl}(\mathbf{r}_{h1})\\ \psi_{ei}(\mathbf{r}_{h2}) & \psi_{ej}(\mathbf{r}_{h2}) & \psi_{hk}(\mathbf{r}_{h2}) & \psi_{hl}(\mathbf{r}_{h2})\end{vmatrix}, \tag{5}$$

[a] Electronic mail: klenovsky@physics.muni.cz

We require that the trial wavefunctions defined in the Eqs. (2-5) be normalized, by imposing that $\sum_m |\eta_m|^2 = 1$.

In all generality, for any total number of fermions N in the system, the m-th Slater determinant is written as:

$$\left|D_m^M\right\rangle = \frac{1}{\sqrt{N!}} \sum_P (-1)^P \phi_{P(i_1)}(\mathbf{r}_1)\phi_{P(i_2)}(\mathbf{r}_2)\dots\phi_{P(i_N)}(\mathbf{r}_N), \tag{6}$$

where $N \equiv N_e + N_h$, with N_e (N_h) the number of electrons (holes) in the complex M (e.g., $N_e = 2$, $N_h = 1$ for the negative trion X^-). The hole and electron wavefunctions are joined in a unique set $\{\phi_1, \dots, \phi_{N_h+N_e}\} \equiv \{\psi_{e_1}\dots\psi_{e_{N_e}}, \psi_{h_1}\dots\psi_{h_{N_h}}\}$, as well as their positions $\mathbf{r}$, for the sake of notational convenience.

The Hamiltonian may be written as

$$\begin{aligned}\hat{H}^M = \hat{H}_0^M + \hat{V}^M &= \hat{H}_0^M + \sum_{\substack{s,t\in\{1\dots N\}\\ s<t}} \frac{q_s q_t}{4\pi\epsilon|\mathbf{r}_s - \mathbf{r}_t|} \\ &= \hat{H}_0^M + \sum_{\substack{s,t\in\{1\dots N\}\\ s<t}} \hat{V}(\mathbf{r}_s, \mathbf{r}_t),\end{aligned} \tag{7}$$

where $\hat{H}_0^M$ is the non-interacting part of the Hamiltonian, $\varepsilon(\mathbf{r})$ and ε_0 are the relative and the vacuum permittivities, respectively, and $q_s, q_t \in \{-e, +e\}$ where e is the elementary charge. The matrix elements of the Hamiltonian in the basis of the Slater determinants are thus given by

$$\left\langle D_n^M \middle| \hat{H}^M \middle| D_m^M \right\rangle = \left\langle D_n^M \middle| \hat{H}_0^M \middle| D_m^M \right\rangle + \left\langle D_n^M \middle| \hat{V}^M \middle| D_m^M \right\rangle. \tag{8}$$

The matrix elements of the Coulomb interaction take the following form:

$$\begin{aligned}\left\langle D_n^M \middle| \hat{V}^M \middle| D_m^M \right\rangle &= \frac{1}{N!}\sum_{PQ}(-1)^{P+Q} \sum_{\substack{s,t\in\{1\dots N\}\\ s<t}} \\ &\int\dots\int \mathrm{d}\mathbf{r}_1\dots\mathrm{d}\mathbf{r}_N \phi^*_{P(i_1)}(\mathbf{r}_1)\dots\phi^*_{P(i_N)}(\mathbf{r}_N) V(\mathbf{r}_s,\mathbf{r}_t)\phi_{Q(j_1)}(\mathbf{r}_1)\dots\phi_{Q(j_N)}(\mathbf{r}_N) \\ &= \sum_{\substack{s,t\in\{1\dots N\}\\ s<t}} \sum_P (-1)^P \int \Bigg(\prod_{i\notin\{s,t\}} d\mathbf{r}_i\Bigg) \phi^*_{i_1}(\mathbf{r}_1)\phi_{P(j_1)}(\mathbf{r}_1)\dots\phi^*_{i_{s-1}}(\mathbf{r}_{s-1})\phi_{P(j_{s-1})}(\mathbf{r}_{s-1}) \\ &\phi^*_{i_{s+1}}(\mathbf{r}_{s+1})\phi_{P(j_{s+1})}(\mathbf{r}_{s+1})\dots\phi^*_{i_{t-1}}(\mathbf{r}_{t-1})\phi_{P(j_{t-1})}(\mathbf{r}_{t-1})\phi^*_{i_{t+1}}(\mathbf{r}_{t+1})\phi_{P(j_{t+1})}(\mathbf{r}_{t+1}) \\ &\dots\phi^*_{i_N}(\mathbf{r}_N)\phi_{P(j_N)}(\mathbf{r}_N) \times \int d\mathbf{r}_s d\mathbf{r}_t \phi^*_{i_s}(\mathbf{r}_s)\phi^*_{i_t}(\mathbf{r}_t)V(\mathbf{r}_s,\mathbf{r}_t)\phi_{P(j_s)}(\mathbf{r}_s)\phi_{P(j_t)}(\mathbf{r}_t) \\ &= \sum_{\substack{s,t\in\{1\dots N\}\\ s<t}} \sum_P (-1)^P \delta_{i_1,P(j_1)}\dots\delta_{i_{s-1},P(j_{s-1})}\delta_{i_{s+1},P(j_{s+1})}\dots\delta_{i_{t-1},P(j_{t-1})} \\ &\delta_{i_{t+1},P(j_{t+1})}\dots\delta_{i_N,P(j_N)} \times \int d\mathbf{r}_s d\mathbf{r}_t \phi^*_{i_s}(\mathbf{r}_s)\phi^*_{i_t}(\mathbf{r}_t)V(\mathbf{r}_s,\mathbf{r}_t)\phi_{P(j_s)}(\mathbf{r}_s)\phi_{P(j_t)}(\mathbf{r}_t).\end{aligned} \tag{9}$$

We introduce the sets

$$\begin{aligned}&S_n \equiv \{i_1, \dots i_N\},\ S_m \equiv \{j_1, \dots j_N\} \\ &\Delta_{nm} \equiv S_n \setminus S_m,\end{aligned} \tag{10}$$

and consider the different cases, depending on the number of elements of the set Δ_{nm}, noted $\mathbf{card}(\Delta_{nm})$. Eq. (9) implies that $\mathbf{card}(\Delta_{nm}) > 2 \Rightarrow \left\langle D_n^M \middle| \hat{V}^M \middle| D_m^M \right\rangle = 0$.

Conversely, if $\mathbf{card}(\Delta_{nm}) = 2$, then we may denote $\begin{cases}\Delta_{nm} = \{i,j\}, & \text{with } i<j \\ \Delta_{mn} = \{k,l\}, & \text{with } k<l,\end{cases}$

so that

$$\begin{aligned}\left\langle D_n^M \middle| \hat{V}^M \middle| D_m^M \right\rangle &= \int d\mathbf{r}_1 d\mathbf{r}_2 \phi^*_i(\mathbf{r}_1)\phi^*_j(\mathbf{r}_2)\left(\frac{q_i q_j}{4\pi\epsilon|\mathbf{r}_1 - \mathbf{r}_2|}\right)\phi_k(\mathbf{r}_1)\phi_l(\mathbf{r}_2) \\ &- \int d\mathbf{r}_1 d\mathbf{r}_2 \phi^*_i(\mathbf{r}_1)\phi^*_j(\mathbf{r}_2)\left(\frac{q_i q_j}{4\pi\epsilon|\mathbf{r}_1 - \mathbf{r}_2|}\right)\phi_l(\mathbf{r}_1)\phi_k(\mathbf{r}_2) \\ &= V_{ij,kl} - V_{ij,lk},\end{aligned} \tag{11}$$

where

$$
\begin{aligned}
V_{ij,kl} &\equiv (1-\delta_{ij})(1-\delta_{kl}) \int d\mathbf{r}_1 d\mathbf{r}_2 \phi_i^*(\mathbf{r}_1)\phi_j^*(\mathbf{r}_2)\left(\frac{q_i q_j}{4\pi\epsilon|\mathbf{r}_1-\mathbf{r}_2|}\right)\phi_k(\mathbf{r}_1)\phi_l(\mathbf{r}_2),\\
q_i &= \begin{cases} |q| & \text{if } i \le N_e \\ -|q| & \text{if } i > N_e. \end{cases}
\end{aligned}
\tag{12}
$$

Similarly, if $\mathbf{card}(\Delta_{nm}) = 1$, then we may denote $\begin{cases}\Delta_{nm} = \{i\},\\ \Delta_{mn} = \{k\},\end{cases}$

so that

$$
\left\langle D_n^M \middle| \hat{V}^M \middle| D_m^M \right\rangle = \frac{1}{2}\sum_{j\in S_n} V_{ij,kj} - V_{ij,jk}. \tag{13}
$$

The last case to consider is $\mathbf{card}(\Delta_{nm}) = 0$, which corresponds to the Slater determinant-diagonal matrix elements of the Coulomb interaction. In this case, Eq. (9) becomes

$$
\left\langle D_n^M \middle| \hat{V}^M \middle| D_n^M \right\rangle = \frac{1}{2}\sum_{i,j\in S_n,} V_{ij,ij} - V_{ij,ji}. \tag{14}
$$

Once the non-interacting part of the Hamiltonian is taken into account, Eq.(3) of the main text is obtained.

We further comment on the numerical difficulty arising in the evaluation of the six-fold integral defined in Eq. (12) above. We chose to handle it by using the Green's function method[1,2] as follows:

$$
\begin{aligned}
&\nabla\left[\varepsilon(\mathbf{r})\nabla\hat{U}_{ajl}(\mathbf{r})\right] = \frac{4\pi e^2}{\varepsilon_0}\psi_{aj}^*(\mathbf{r})\psi_{al}(\mathbf{r}),\\
&V_{ij,kl} = \left\langle \psi_{bi} \middle| \hat{U}_{ajl} \middle| \psi_{bk} \right\rangle,
\end{aligned}
\tag{15}
$$

where $a, b \in \{e, h\}$. We note that out of all 64 possible combinations in $\psi_{aj}^*(\mathbf{r})\psi_{al}(\mathbf{r})$ and $\left\langle \psi_{bi} \middle| \hat{U}_{ajl} \middle| \psi_{bk} \right\rangle$, respectively, only eight corresponding to the same Bloch functions on either side of the multiplication need to be calculated. This is a result of the envelope function approach that we have used, see Eq. (1).

We have also taken advantage of the approximation $\varepsilon(\mathbf{r}, \mathbf{r}') \approx \varepsilon(\mathbf{r})$ as in Ref. 3 and, thus, we have not treated the electron-phonon interaction exactly as in, e.g., Ref. 4. However, since we have considered the multi-excitonic complexes in this work only and omitted more subtle effects like, e.g., FSS we find this approximation acceptable. We further note that we have achieved numerically effective evaluation of Eq. (15) by LU decomposition of the Poisson's differential operator using the iterative SuperLU algorithm, see Ref. 5. We have coded CI in the Python 2.7 programming language[6].

SII. PHOTOLUMINESCENCE OF INAS/GAAS QDS

The pumping power dependence of PL from type-I InAs/GaAs QDs is shown in Fig. S1. Note particularly that the energy of X^0 does not change with P, i.e. $\Delta E \approx 0$. On the other hand large blue-shift of XX^0 with P is due to the structure of the sample. It is a stack of 3 layers of InAs QDs grown above each other. Due to that XX^0 in Fig. S1 originates in transitions when the quasi-particles are located in different QDs of the multilayer and are thus of type-II displaying considerable blue-shift with P. We note that biexcitons originating in transitions from one QD cannot be resolved by our PL measurements.

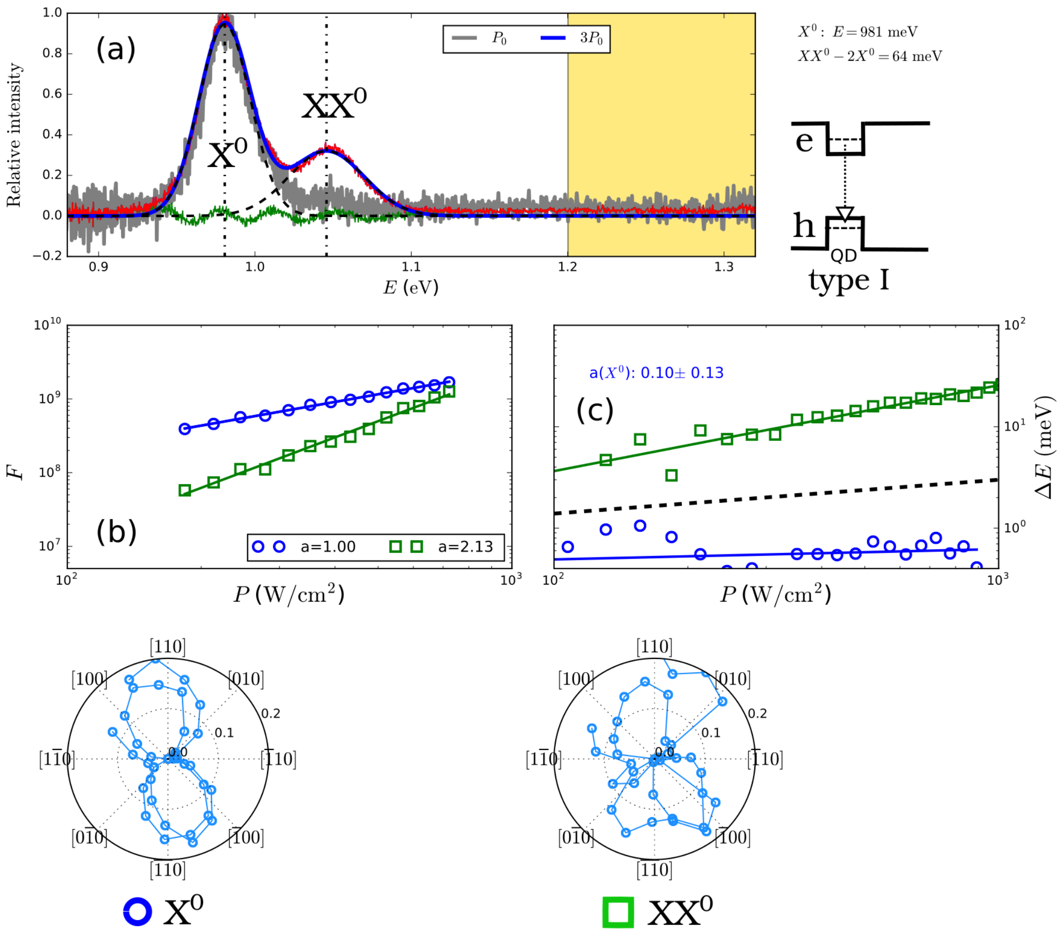

Figure S1. (color online) (a) PL spectra of InAs/GaAs type-I QDs measured for two pumping powers P differing by a factor of three (grey and red curves). The fit by the sum of Gaussian curves is shown for $3P_0$ (blue curve) and the individual bands corresponding to X^0 and XX^0 transitions are shown by broken curves, respectively. The difference between data and fit is given by green curve. We show by the dotted vertical lines the energy of the bands for $3P_0$ in order to facilitate the comparison with those for P_0. The inset next to panel (a) shows the spectral position of each band and a schematic band diagram of the recombination pattern (not in scale). In (b) we show the P-dependence of the oscillator strength F of the identified bands in log-log scale and their fits by linear lines, respectively, for X^0 (blue circles) and XX^0 (green squares), respectively. The slopes a of the fitted lines are given in the inset of panel (b) and for more clarity were normalized so that $a = 1$ for X^0. Panel (c) depicts the change of the emission energy ΔE with P in log-log scale. The designation is the same as in (b) and the data were fitted by a single linear function, the fitted slope a of that for X^0 being given in the inset. The $\Delta E \sim P^{1/3}$ dependence[7] is shown by broken curve. The polar graphs at the bottom show $C(\alpha)$ of individual identified bands.

SIII. EXPERIMENTAL RESULTS ON A VARIETY OF SAMPLES

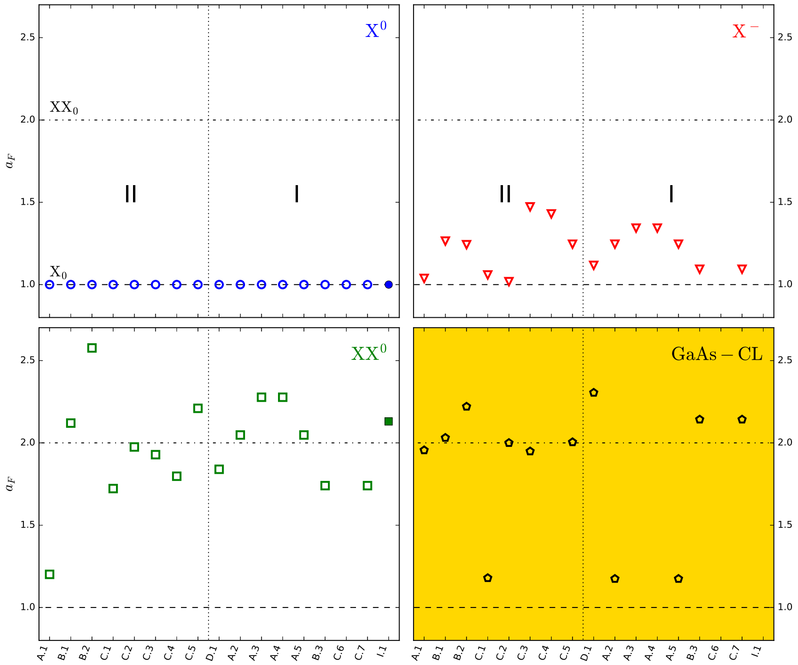

Figure S2. (color online) Summary of slopes a_F of the linear fit of $F(P)$ for X^0 (blue circles), X^- (red triangles), XX^0 (green squares), and that for the transition between bulk GaAs and CL (black pentagons). The data are shown for four samples with InGaAs/GaAsSb/GaAs QDs, marked as A through D on the horizontal axis and the sample with InAs/GaAs QDs marked by I; different numbers correspond to different position on the corresponding sample. The slopes a_F were normalized so that $a_F = 1$ for X^0. The dotted vertical line divides QDs corresponding to type-II and type-I confinement, respectively. The horizontal lines mark the values of the slopes for free non-interacting exciton (broken) and free non-interacting biexciton (dotted).

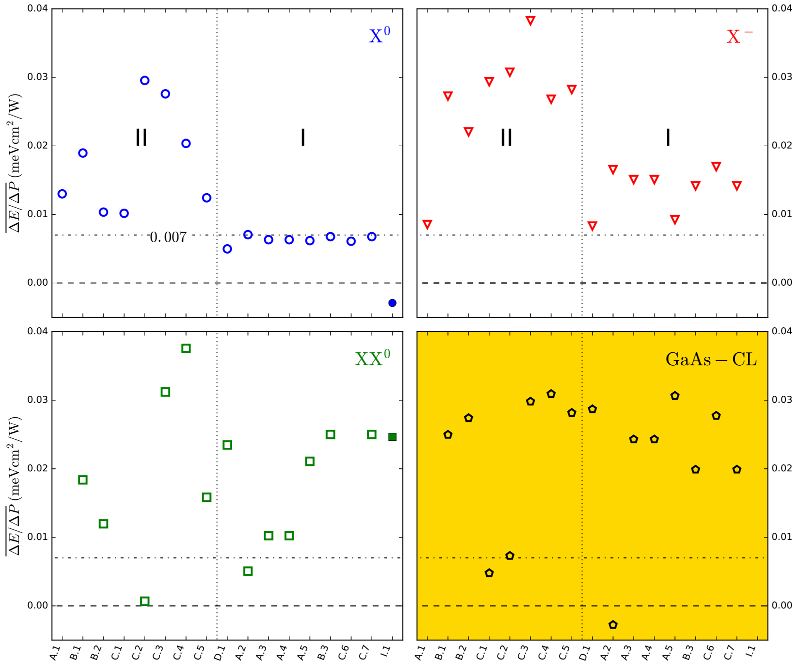

Figure S3. (color online) Summary of the mean blue-shifts $\overline{\Delta E/\Delta P}$. The marking of the data is the same as in Fig. S2 except for the broken horizontal line marking zero value and the dotted horizontal one marking energy shift of $\overline{\Delta E/\Delta P} = 0.007$ according to which we distribute the QD samples to those having type-I (or q-type-I) confinement and those presenting with type-II transition, respectively.

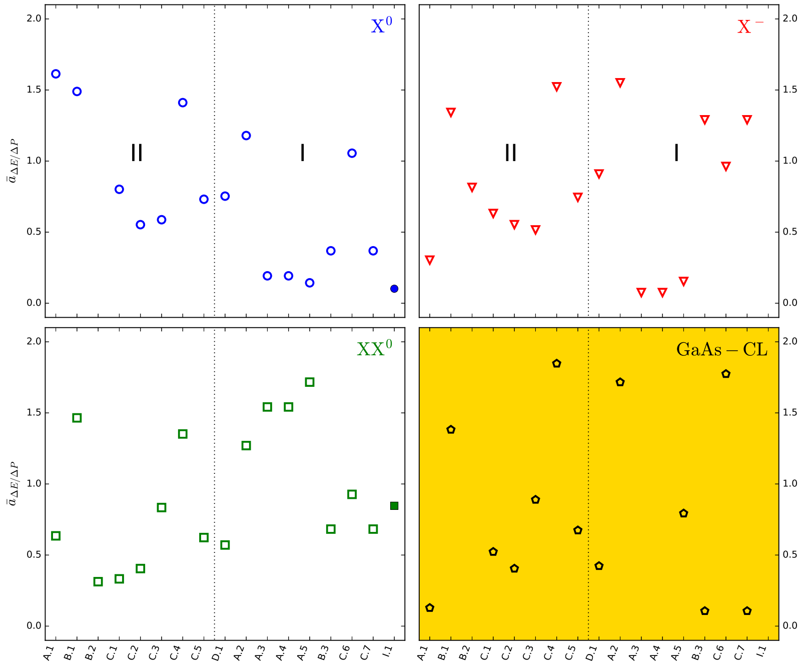

Figure S4. (color online) Summary of the mean exponents $\bar{a}_{\Delta E/\Delta P}$ of $\Delta E = bP^a$ dependence used for fitting of the $E(P)$ data. The marking is the same as in Fig. S2 except for the the absence of the horizontal lines.

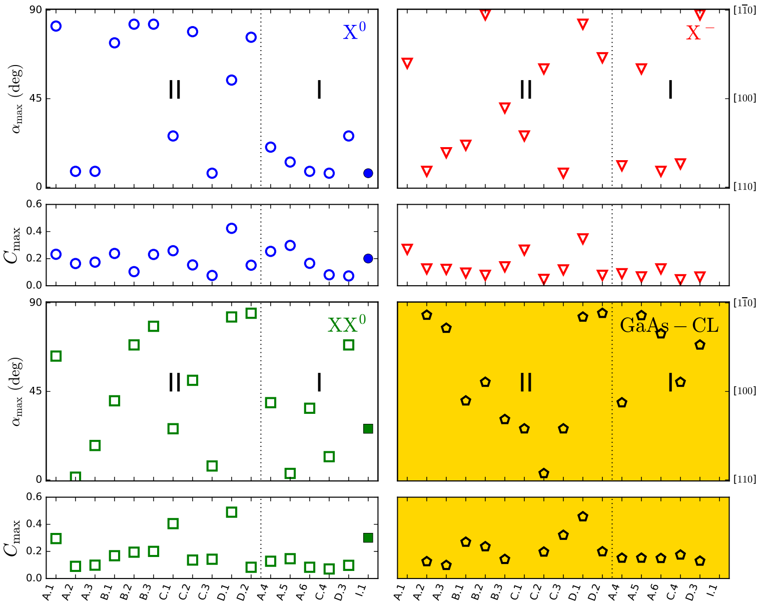

Figure S5. (color online) Summary of the azimuth $\alpha_{\max}$ and degree $C_{\max}$ of the polarization anisotropy of the multi-particle bands. The marking of the data is the same as in Figs. S2. For each complex the upper panel corresponds to $\alpha_{\max}$ and the lower one to $C_{\max}$.

SIV. BLUE-SHIFT WITH PUMPING

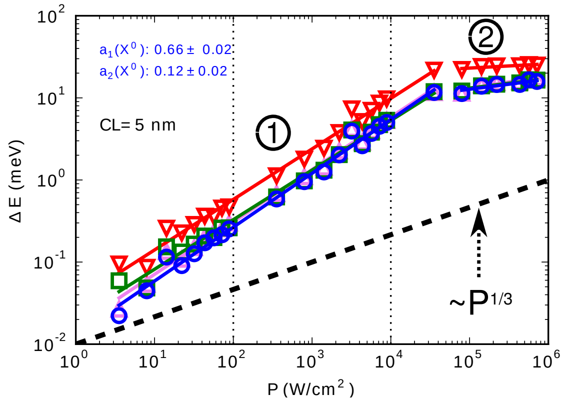

Figure S6. (color online) Energy shift ΔE with P for CL thicknesses of 5 nm. The results are shown for X^0 (blue circles), X^+ (magenta upward triangles), X^- (red downward triangles), and XX^0 (green squares). The numbers 1 and 2 depict the different slopes.

REFERENCES

[1] A. Schliwa, M. Winkelnkemper, and D. Bimberg, Physical Review B **79**, 075443 (2009).
[2] O. Stier, A. Schliwa, R. Heitz, M. Grundmann, and D. Bimberg, Physica Status Solidi B-basic Research **224**, 115 (2001).
[3] O. Stier, M. Grundmann, and D. Bimberg, Phys. Rev. B **59**, 5688 (1999).
[4] T. Takagahara, Phys. Rev. B **62**, 16840 (2000).
[5] X. Y. S. Li, Acm Transactions On Mathematical Software **31**, 302 (2005).
[6] Python Software Foundation. Python Language Reference, version 2.7. Available at http://www.python.org.
[7] F. Hatami, M. Grundmann, N. N. Ledentsov, F. Heinrichsdorff, R. Heitz, J. Bohrer, D. Bimberg, S. S. Ruvimov, P. Werner, V. M. Ustinov, P. S. Kop'ev, and Z. I. Alferov, Phys. Rev. B **57**, 4635 (1998).
